# Human group 2 innate lymphoid cells do not express the IL-5 receptor

**DOI:** 10.1016/j.jaci.2017.04.025

**Published:** 2017-11

**Authors:** Adam K.A. Wright, Cathryn Weston, Batika M.J. Rana, Christopher E. Brightling, David J. Cousins

**Affiliations:** a Department of Infection, Immunity and Inflammation, University of Leicester, Leicestershire, United Kingdom; b MRC &Asthma UK Centre in Allergic Mechanisms of Asthma, King's College London, London, United Kingdom; c MRC Laboratory of Molecular Biology, Cambridge, United Kingdom; d Institute of Lung Health, NIHR Leicester Respiratory Biomedical Unit, University Hospitals of Leicester NHS Trust, Leicestershire, United Kingdom

To the Editor:

Eosinophils, cardinal effector cells of type 2 inflammation, contribute to the clinical and immunopathologic manifestations of asthma^1^ and chronic obstructive pulmonary disease^2^ inflammatory endotypes. Eosinophil biology is governed by IL-5, a cytokine that binds with high affinity to a specific IL-5 receptor α subunit (IL-5Rα) before forming a heterodimeric receptor complex with the β subunit.^3^ IL-5 signaling promotes differentiation, maturation, and survival of eosinophil-committed progenitors while acting on mature eosinophils to enhance their migratory potential and effector responses.^4^ IL-5 signaling also promotes alternative splicing of the IL-5Rα gene to generate transmembrane forms of IL-5Rα.^5^ Because the IL-5-IL-5R axis appears to be restricted to eosinophils and basophils (and their progenitors),^6^ therapeutic regulation of these cells through the neutralization of circulating IL-5 (eg, mepolizumab and reslizumab)^1^ or IL-5Rα ligation (eg, benralizumab),^1^, ^2^ have emerged as effective strategies to deplete blood, tissue, and airway eosinophils and consequently reduce exacerbation rates and improve lung function.^1^

Basophils and group 2 innate lymphoid cells (ILC2s) are important innate sources of type 2 cytokines, including IL-5, in response to epithelial-derived cytokines such as IL-33.^7^, ^8^ ILC2s, however, have emerged as central and critical innate coordinators of steady-state eosinophilopoiesis and epithelial cell–driven, type 2 immunopathology in asthma.^8^ As an important upstream regulator of eosinophil function we asked whether ILC2s, like their basophil counterparts, express the IL-5Rα subunit because this may have important implications for our understanding of the role of the IL-5-IL-5R axis in disease and therapeutic targeting of these rare but important innate immune cells. Detailed methods are provided in this article's Online Repository at www.jacionline.org.

ILC2s are rare innate lymphocytes that lack the T-cell receptor complex and all known lineage markers but, similar to other type 2 cytokine-producing cells such as T_H_2 cells, eosinophils, and basophils, express the type 2 prostaglandin D_2_ receptor, DP2/CRT_H_2 (CD294).^9^ We defined blood ILC2s as cells with singlet, lymphocyte light scatter properties (Fig 1, *A* and *B*), lineage (CD2, 3, 14, 16, 19, 56, and 235a)^−^ but CD294^+^ (Fig 1, *C*). A large proportion of cells within this gate were basophils, (CD123^+^ cells in Fig 1, *D*); however, CD294^+^, CD123^−^ ILC2s were present (Fig 1, *D*, and light scatter in *E*). Basophils displayed a higher level of CD125-phycoerythrin (PE) staining than did the ILC2s (Fig 1, *D*) that could be blocked in the presence of rhIL-5 (Fig 1, *F*), confirming the specificity of the antibody and basophil IL-5Rα expression. In contrast to basophils, there was no change in CD125-PE signal intensity when ILC2s were incubated with rhIL-5, indicating that there was no IL-5Rα expression on these cells (Fig 1, *F*). To confirm the reproducibility of these findings, we recruited 6 control and 13 volunteers with asthma (see clinical details in Table E1 in this article's Online Repository at www.jacionline.org) and measured IL-5Rα expression. The total mean (SD) % of ILC2s within the lymphocyte gate for both control and asthma groups were 0.1% ± 0.1% and 0.03% ± 0.03%, respectively (*P* = .06, Mann-Whitney). In total, combining asthma and control data sets, the basophil CD125 geometric mean fluorescence intensity (GMFI) (mean ± SD) in the absence or presence of rhIL-5 was 1285 ± 614 and 401 ± 315 respectively, representing a significant reduction in GMFI (Fig 1, *G* and *I*). In contrast, the ILC2 CD125 GMFI (mean ± SD) in the absence or presence of rhIL-5 was 45 ± 45 and 42 ± 33, respectively (Fig 1, *H* and *I*). In a subset of samples (n = 3), where basophils and ILC2s could be detected in whole blood, data were qualitatively similar. There were no significant differences between the control and asthma subjects for any of the flow cytometric measurements obtained.

**Fig 1:**
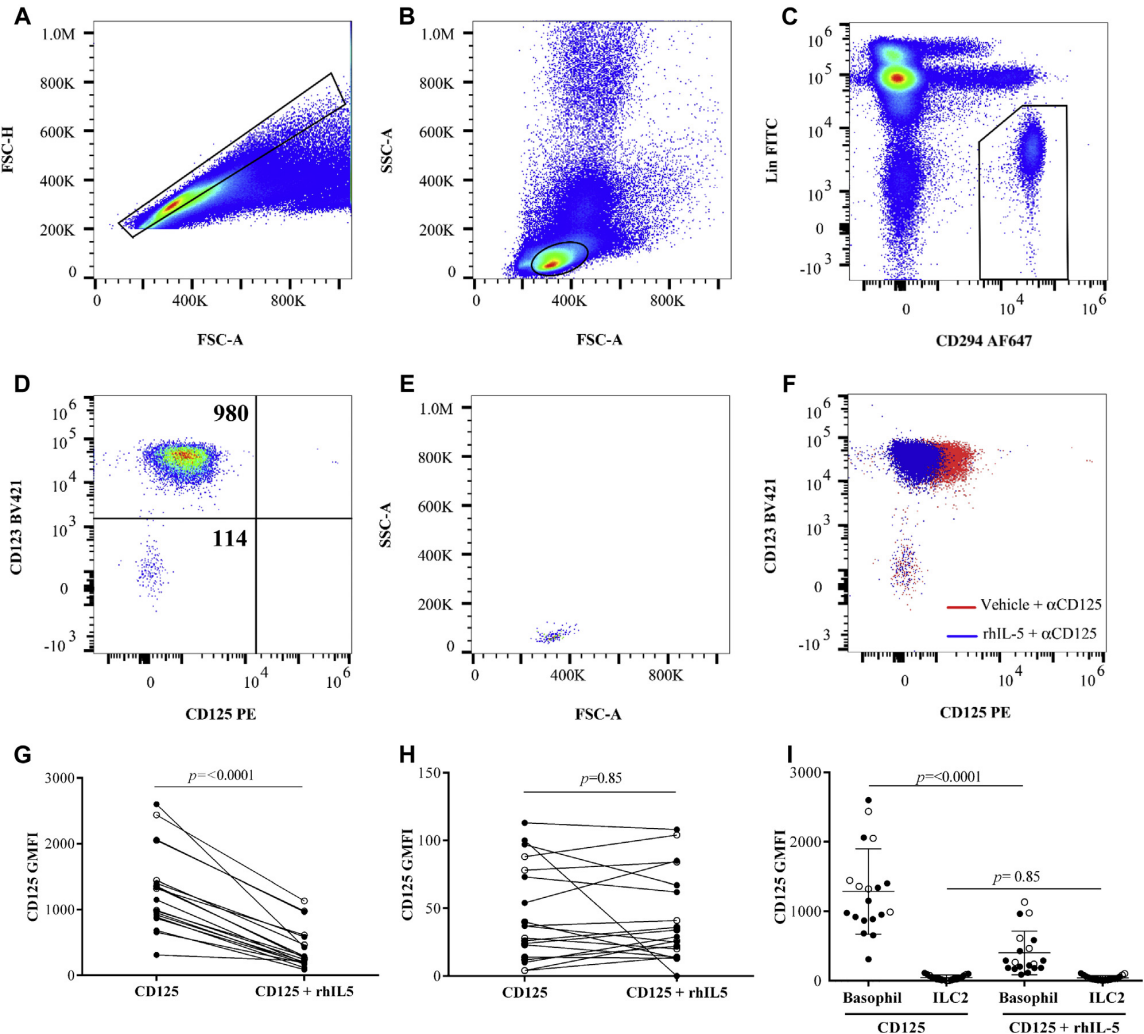
CD125 protein is absent on blood ILC2s. **A,** Total PBMCs, singlets within boxed region. **B,** Singlet light scatter with lymphocyte gate overlaid. **C,** Lineage^−^, CD294^+^ cells were identified (boxed region) encompassing **(D)** CD123^+^ basophils and CD123^−^ ILC2s, in the upper and lower left quadrant, respectively, with CD125 GMFI values. **E,** ILC2 light scatter properties. **F,** Overlay of basophil and ILC2 CD125-stained cells in the absence (red) or presence (blue) of rhIL-5. **(G)** Basophil and **(H)** ILC2 CD125 GMFI paired data (mean ± SD) from 6 healthy volunteers (open) and 13 (filled) volunteers with asthma, also shown in **(I)** unpaired on the same axes. Statistical comparisons were made using a Wilcoxon matched-pairs signed rank test. *APC*, Allophycocyanin; *FITC*, fluorescein isothiocyanate; *FSC-A*, forward scatter-area; *FSC-H*, forward scatter-height; *SSC-A*, side scatter-area.

We next sought to confirm whether the IL-5Rα data were reflected at the RNA level using real-time PCR probes spanning the exon boundaries present within IL-5Rα subunit variants (see Fig E1, *A*, in this article's Online Repository at www.jacionline.org). For this, ILC2s were isolated from 3 additional donors and cultured in the absence or presence of cytokines known to enhance survival (IL-2 and IL-7) and activate ILC2s (IL-25 and IL-33). RNA was isolated at 6 time points (day 0, 1, 2, 4, 7, and 14) and converted into cDNA. Eosinophil cDNA was included as a positive control. The IL-5Rα transcripts were not detected in ILC2s at any time point; however, an increase in IL-5 mRNA was observed, indicating that the culture conditions were sufficient to activate the cells (Fig E1, *B* and *C*).

Finally, using a similar approach to the blood analyses (Fig 1) and using surgically removed lung tissue from 7 subjects (see Table E2 in this article's Online Repository at www.jacionline.org), we asked whether tissue-derived ILC2s and basophils expressed IL-5Rα. Tissue-derived basophils were defined as viable, CD45^+^Lin^−^CD294^+^CD123^+^CD127^−^ cells and ILC2s as viable, CD45^+^Lin^−^CD294^+^CD123^−^CD127^+^ cells present within the singlet lymphocyte gate (Fig 2, *A*-*E*). Basophil CD125-PE GMFI (mean ± SD, 612 ± 327) could be reversed in the presence of rhIL-5 to a mean of 317 ± 91 (example in Fig 2, *F*, and cumulative data in Fig 2, *H*). Using the super enhanced Dmax (SED) algorithm to compare basophil populations stained in the absence or presence of IL-5 for each of the 7 donors revealed that 71.1% ± 4.2% basophils were CD125 positive (Fig 2, *I*). In contrast, ILC2 CD125 GMFI (mean ± SD) was significantly lower than that of basophils and showed little change in the presence of rhIL-5 (190 ± 42 and 145 ± 49, respectively) (example in Fig 2, *G*, and cumulative data in Fig 2, *H*). SED analysis of ILC2s stained in the absence or presence of rhIL-5 for each of the 7 donors (Fig 2, *I*) did not reveal a CD125-positive subset (mean ± SD, 3.3% ± 0.89%). Data from these 7 subjects consistently show that tissue-derived basophils express the IL-5Rα whereas tissue-derived ILC2s do not (Fig 2, *H*).

**Fig 2:**
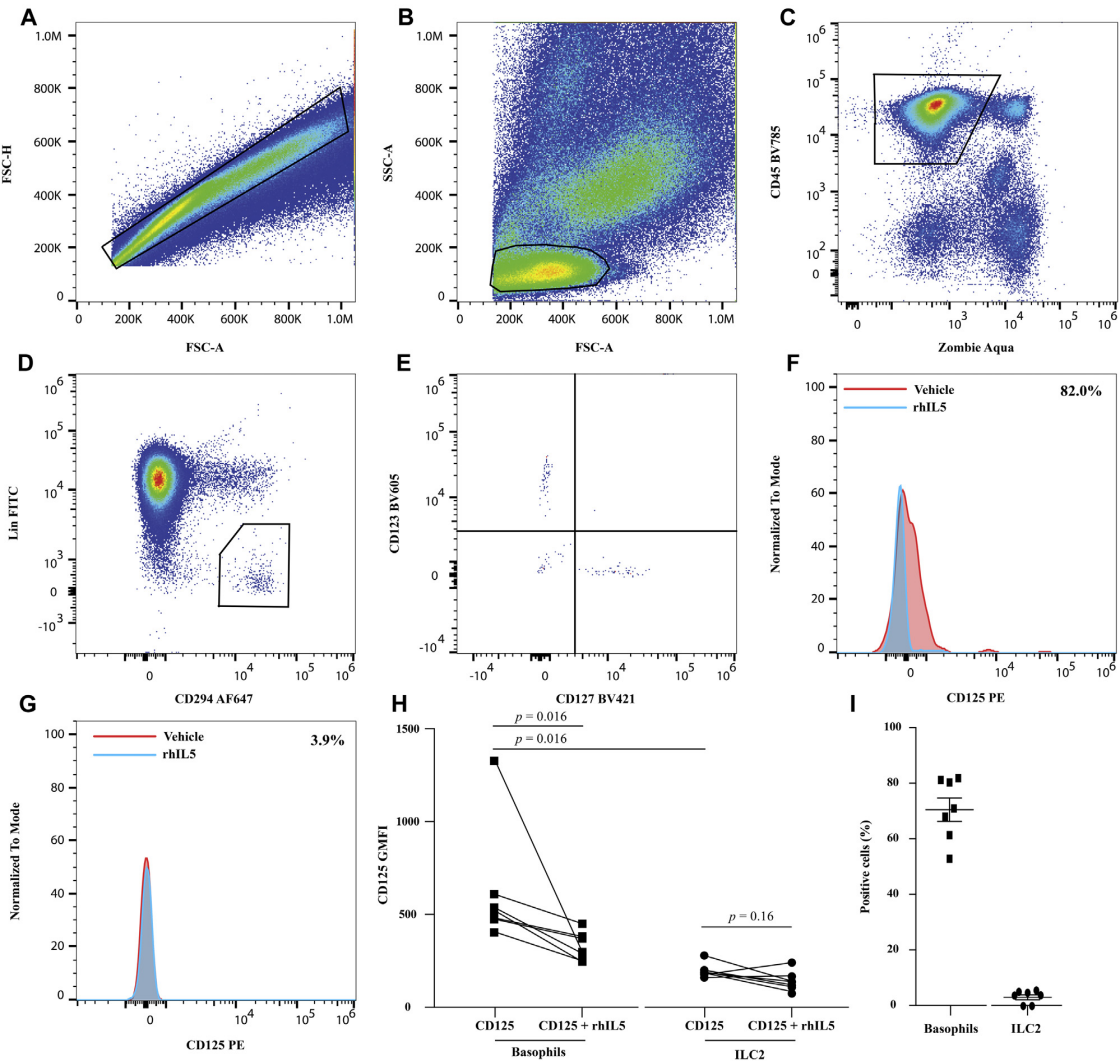
CD125 protein is absent on lung-derived ILC2s. **A,** Total lung tissue cells, singlets within boxed region. **B,** Singlet light scatter properties with lymphocyte gate overlaid. **C,** Viable CD45^+^ cells (boxed region). **D,** Lineage^−^ CD294^+^ cells (boxed region). **E,** ILC2s (CD123^−^CD127^+^) and basophils (CD123^+^CD127^−^) were identified. **(F)** Basophil CD125 fluorescence and **(G)** ILC2 CD125 fluorescence in the absence (red) or presence of rhIL-5 (blue). **H,** Collated basophil and ILC2 CD125 GMFI (mean ± SD) data (n = 7). **I,** Proportion of CD125^+^ basophils and ILC2s (calculated using SED); exemplar values given in Fig 2, *F* and *G*. Statistical comparisons were made using a Wilcoxon matched-pairs signed rank test. *FITC*, Fluorescein isothiocyanate; *FSC-A*, forward scatter-area; *FSC-H*, forward scatter-height; *SSC-A*, side scatter-area.

The strength(s) of this study lies in the fact that we have measured protein IL-5Rα subunit expression in a rare critical regulator of type 2 inflammation, ILC2s, and in the context of rhIL-5–mediated receptor blockade/downregulation, rather than an isotype control, which is less robust. The robustness of our data using human peripheral blood (from donors with asthma), *ex vivo* activated cells, and lung tissue cells diminishes the likelihood that IL-5Rα–expressing ILC2s are present in asthmatic tissue. Although it would be desirable to extend our blood and tissue observations to investigate whether ILC2s in bronchial biopsies from patients with asthma express IL-5Rα, the paucity of tissue ILCs and the need for multiple immunological markers to positively identify them severely limits this approach. These results extend the list of cells^6^ that are known not to express the IL-5Rα subunit, specific for the biological activities of IL-5. Moreover, we show that rare tissue-derived basophils, like their blood counterparts, express the IL-5Rα. Our data suggest that the success of therapeutic interventions targeting IL-5/R is unlikely to be mediated directly on ILC2s but may function via both eosinophils and basophils.

## Figures and Tables

**Fig E1:**
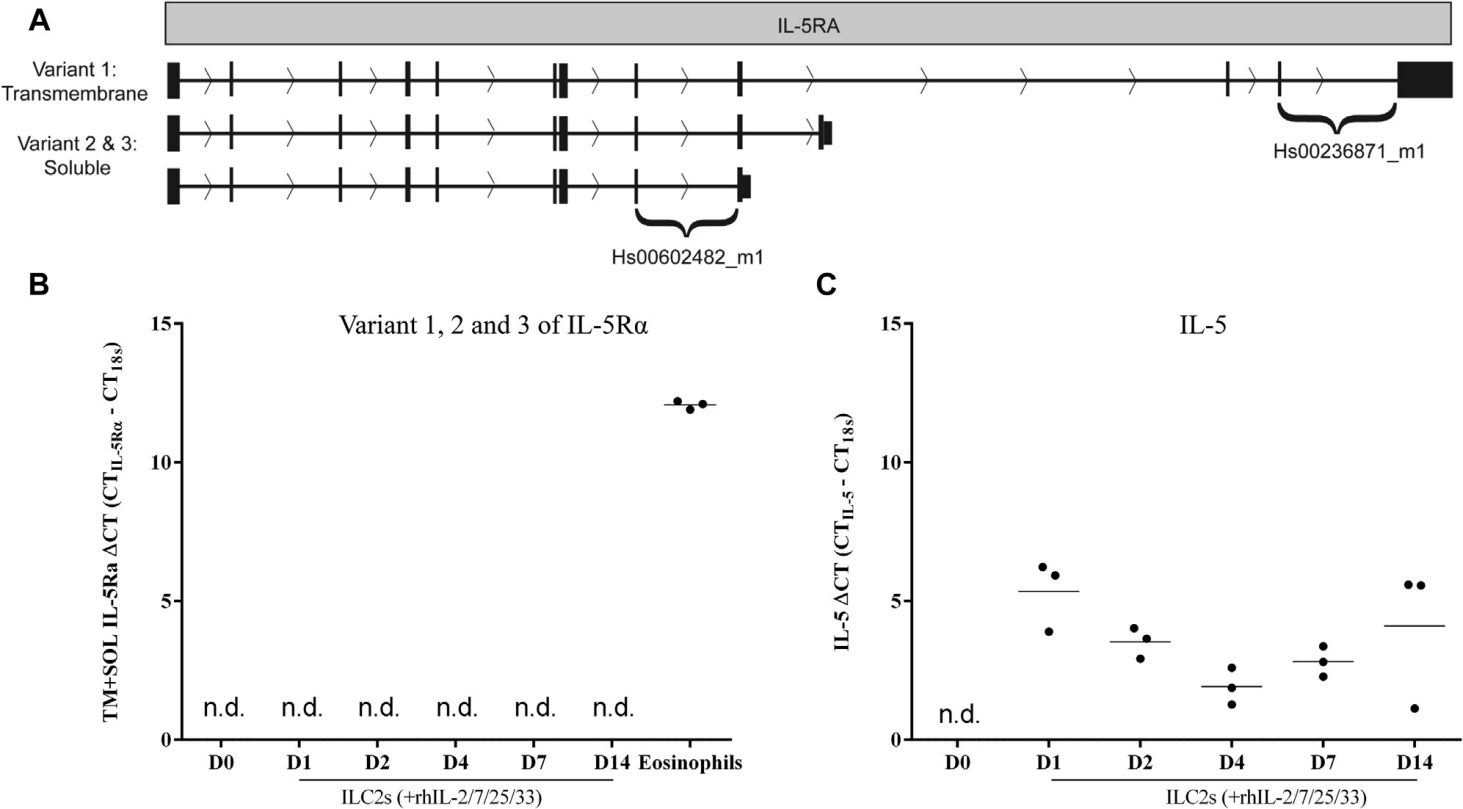
ILC2s do not express IL-5Rα transcripts. **A,** Schematic representation of IL-5Rα mRNA showing the splice variants and probe sets Hs00602482_m1 (detecting soluble and transmembrane variants) and Hs00236871_m1 (transmembrane only) used in this study. ΔCT values were calculated relative to 18S mRNA for **(B)** all IL-5Rα transcript variants using the Hs00602482_m1 probe and **(C)** IL-5 transcripts using Hs00174200_m1. In Fig E1, *B* and *C*, data are derived from isolated ILC2s (n = 3) that were grown for up to 14 days in the presence of rhIL-2, rhIL-7, rhIL-25, and rhIL-33. In Fig E1, *B*, cDNA generated from freshly isolated eosinophils was also included as a positive control in each IL-5Rα assay. In Fig E1, *C*, IL-5 transcript data are shown to confirm activation in the presence of cytokines; low ΔCT values reflect high abundance of the IL-5 transcript. *n.d.*, Not detected.

**Table E1:** Clinical characteristics of the study participants recruited for blood collection

Characteristic	Control	Asthma	*P* value∗
n	6	13	
Age (y)†	50 ± 15	59 ± 13	.18
Sex: M:F	4:2	6:7	.63
Smoking history (never, ex, current)‡	5, 1, 0	9, 4, 0	1.0
BMI (kg/m^2^)†	27 ± 4	29 ± 6	.47
Post-BD FEV_1_ (L)†	3.6 ± 0.8	2.5 ± 0.9	.02
Post-BD FEV_1_ (% predicted)†	109 ± 16	92 ± 24	.17
FEV_1_/FVC (ratio)†	0.82 ± 0.06	0.71 ± 0.09	.01
WBC (×10^9^/L)†	6.4 ± 0.9§	7.1 ± 2.8‖	.63
Eosinophils (×10^9^/L)†	0.16 ± 0.04§	0.28 ± 0.16‖	.12
Eosinophil (%)	2.5 ± 0.6§	4.3 ± 2.4‖	.13
Basophils (×10^9^/L)†	0.04 ± 0.02§	0.04 ± 0.02‖	.97
GINA 1, 2, 3, 4, 5	NA	1, 1, 0, 7, 4	NA
Beclomethasone dipropionate equivalents (μg)¶	NA	1000 (650-1600)	NA

*BD*, Bronchodilator; *BMI*, body mass index; *F*, female; *FVC*, forced vital capacity; *GINA*, Global Initiative for Asthma; *M*, male; *NA*, not applicable; *WBC*, white blood cell.

∗ Unpaired *t* test.

† Mean ± SD.

‡ All ex-smokers have quit smoking for 3+ years.

§ n = 5.

‖ n = 12.

¶ Median (interquartile range).

**Table E2:** Clinical characteristics of the study participants recruited for lung tissue collection

Characteristic	Control
n	7
Age (y)∗	66 ± 9
Sex: M:F	4:3
Smoking history (never, ex, current)†	0, 4, 3
WBC (×10^9^/L)∗	9.5 ± 3.7
Eosinophils (×10^9^/L)∗	0.24 ± 0.18
Eosinophil (%)∗	2.4 ± 1.1
Basophils (×10^9^/L)∗	0.04 ± 0.03
Diagnosis	Adenocarcinoma (n = 4) Squamous cell carcinoma (n = 2) Lung volume reduction surgery (n = 1)

*F*, Female; *M*, male; *WBC*, white blood cell.

∗ Mean ± SD.

† All ex-smokers have quit smoking for 2+ years.

## Methods

### Subjects

Control subjects (without a history of respiratory disease) and subjects with an asthma diagnosis were recruited at University Hospitals of Leicester NHS Trust, Leicester, United Kingdom (UK), and provided informed written consent, under ethics (REC reference no. 08/H0406/189) approved by the Leicestershire, Northamptonshire, and Rutland ethics committee, to take part in this study. The recruited subjects with asthma were mostly diagnosed with late-onset asthma. Lung resection material was ethically obtained at University Hospitals of Leicester NHS Trust, under the auspices of the Midlands lung tissue consortium (REC reference no. 07/MRE08/42), from a separate cohort of patients undergoing lung surgery. Clinical characteristics can be found in Table E1 (blood) and Table E2 (lung tissue). Additional control subjects (without a history of respiratory disease) were also recruited under ethical approval from the research ethics committee of Guy's Hospital, London, UK (REC reference no. 09/H804/077) as described previously^E1^ to isolate ILC2 cDNA for downstream experiments.

### Cells

Human eosinophils were purified from whole blood using CD16 microbead depletion of neutrophils over CS columns (both Miltenyi Biotec, Surrey, UK) to a purity of 97% as described previously.^E2^ Purified eosinophils were immediately placed into Qiazol (Qiagen, Manchester, UK) for downstream RNA isolation and used as a positive control for the detection of transmembrane and soluble mRNA variants of the IL-5Rα gene. ILC2s were isolated from peripheral blood by flow cytometric cell sorting and cultured *in vitro* as described in detail previously.^E1^ Briefly, ILC2s were grown at 37°C, 5% CO_2_, in a humidified incubator at a density of 10,000 cells/mL in RPMI 1640 supplemented with 10% FBS, glutamine, penicillin, and streptomycin (all Thermofisher, Paisley, UK). Recombinant cytokines were added as described in the figure legend (rhIL-2 [50 U/mL; Eurocetus, Amsterdam, The Netherlands], rhIL-7 [50 ng/mL; R&D Systems, Abingdon, UK], rhIL-25 [125 ng/mL; R&D Systems], and rhIL-33 (50 ng/mL; R&D Systems]). Lung tissue cells were obtained from enzymatically digested lung obtained within 1 hour of resection (n = 7). Lung tissue bathed in media (Dulbecco modified Eagle medium, with 2% FBS) was cut into small pieces and enzymatically digested using hyaluronidase (0.75 mg/mL final, H3506, Sigma, Gillingham, Dorset, UK) and collagenase (0.75 mg/mL final, C2674, Sigma) for 75 minutes at 37°C. Digested tissue was passed through a syringe, filtered through a 100- and 50-μm gauze, then washed twice with media (centrifuging at 230*g* for 8 minutes, 4°C). Cell counts were performed using hemocytometer and Kimura stain, washed again, and the cell suspension was prepared for flow cytometry, as described below.

### Flow cytometry

PBMCs were isolated following centrifugation of whole blood over Lymphoprep (Stem Cell Technologies, Cambridge, UK). PBMCs were washed in PBS containing 2% FBS (Thermofisher) and 2 mM EDTA (Thermofisher) and the cell density was adjusted to 1 × 10^6^/100 μL before cell surface staining. For lung tissue cells, at least 5 × 10^5^ cells were resuspended in PBS and stained with Zombie Aqua fixable viability kit (as described by the manufacturer [Biolegend, London, UK]) for 20 minutes at room temperature. Following incubation, lung tissue cells were washed and resuspended in 50 μL brilliant violet stain buffer (Becton Dickinson, Oxford, UK) before cell surface staining. A total of 1 × 10^6^ PBMCs or lung tissue cells were then incubated at room temperature for 15 minutes with Human Trustain (Biolegend, London, UK) to block Fc receptors and either a vehicle control (PBS/2%FBS) or rhIL-5 (50 or 100 ng/mL final for PBMCs and lung tissue cells, respectively [Miltenyi Biotec]), to block cell surface staining of IL-5Rα. Cells were then incubated at room temperature with the following anti-human mAbs in a fluorescence minus one^E3^ set-up: BV_421_-conjugated CD123 (clone 6H6, Biolegend), fluorescein isothiocyanate–conjugated lineage (α-CD2, α-CD3, α-CD14, α-CD16, α-CD19, α-CD56, α-CD235a) cocktail (Affymetrix, Cheshire, UK), phycoerythrin-conjugated α-CD125 (clone A14, Becton Dickinson), and AF647-conjugated α-CD294 (clone BM16, Biolegend). In some experiments, whole blood (100 μL) was stained as described above. For single-cell suspensions derived from lung tissue, the lineage cocktail, CD125, and CD294 antibodies (described above) were supplemented with BV_421_-conjugated CD127 (clone A019D5), BV_605_-conjugated CD123 (clone 6H6), and BV_785-_conjugated CD45 (clone HI30) (all Biolegend) to enable identification of tissue-derived ILC2s and basophils. PBMCs were washed with PBS/2%FBS before acquisition, whereas whole blood or lung tissue cells were fixed and lysed (1-step fix and lyse, Affymetrix) to remove erythrocytes according to the manufacturer's protocol. Cell acquisition was performed on an Attune flow cytometer operating NxT software v2.2 (Life Technologies). CD125-PE and rhIL-5 (for blocking purposes) were both titrated in pilot experiments. Compensation beads (Becton Dickinson, High Wycombe, UK) and unstained cells were used to determine compensation settings. Analysis was performed using FlowJo v10 (Tree star, Ashland, Ore).

### RT-PCR

RNA was isolated from purified cell populations using the miRNeasy mini kit (Qiagen), followed by DNAse digestion (Thermofisher) and RNeasy minElute cleanup (Qiagen) according to the respective manufacturer's instructions. RNA integrity was assessed using a Bioanalyser (Agilent, Edinburgh, UK), according to the manufacturer's instructions. For eosinophil samples, cDNA synthesis was performed using superscript II reverse transcriptase (Thermofisher), random hexamers (Roche, West Sussex, UK), and dNTPs (Thermofisher) according to the manufacturer's instructions. Because of low cell numbers, ILC2 cDNA samples were amplified using the Ovation PicoSL WTA system V2 kit (NuGEN, Leek, Netherlands) as per the manufacturer's instructions. Real-time RT-PCR was performed as described previously^E4^ using TaqMan Master mix II (Thermofisher) and TaqMan probe sets Hs00602482_m1 (transmembrane and soluble variants of the IL-5Rα), Hs00236871_m1 (transmembrane only variant of IL-5Rα), or Hs00174200_m1 (IL-5) (Thermofisher) and compared with an 18S rRNA endogenous control (Thermofisher), using FAM-MGB (IL-5Rα probe sets) and VIC-MGB (18S rRNA) dyes, respectively. Cycling conditions were as follows: 50°C for 2 minutes, 95°C for 10 minutes, 95°C for 15 seconds then 60°C for 1 minute for 50 cycles on ViiA7 real-time PCR system (Thermofisher). Because IL-5Rα transcripts were undetectable, to confirm that a sufficient amount of ILC2 cDNA from each condition was present in the reaction, the mean (±SEM) 18S CT values were as follows: D0 (18.4 ± 0.18), D1 (18.5 ± 0.23), D2 (18.0 ± 0.17), D4 (17.4 ± 0.01), D7 (17.4 ± 0.03), D14 (16.8 ± 0.08), eosinophils (16.2 ± 0.4) (representative results from 1 donor). Furthermore, to confirm that ILC2s were activated in the presence of rhIL-2/7/25 and 33, we have presented IL-5 transcript data alongside the IL-5Rα data in Fig E1.

### Statistics

Descriptive and comparative (super enhanced Dmax SED) subtraction % positive^E5^ analyses of flow cytometric population data were performed using FlowJo version 10 (Tree Star). Descriptive and comparative analyses were performed using Graph Pad PRISM v6 (GraphPad software, Inc, La Jolla, Calif), and Shapiro-Wilk normality test was used to determine data distribution. Statistical comparisons were made using paired or unpaired tests where relevant, as indicated, with statistical significance determined if the *P* value was less than .05.
